# Food insecurity among households with children during the COVID-19 pandemic: results from a study among social media users across the United States

**DOI:** 10.1186/s12937-021-00732-2

**Published:** 2021-08-30

**Authors:** Niyati Parekh, Shahmir H. Ali, Joyce O’Connor, Yesim Tozan, Abbey M. Jones, Ariadna Capasso, Joshua Foreman, Ralph J. DiClemente

**Affiliations:** 1 Public Health Nutrition Program, School of Global Public Health, New York University, 715 Broadway New York, Room 1220, New York, 10003 USA; 2 Department of Population Health At NYU Grossman School of Medicine, New York University, New York, USA; 3 Rory Meyers College of Nursing, New York University, New York, USA; 4 Department of Social and Behavioral Sciences, School of Global Public Health, New York University, New York, USA; 5 Global Health Program, School of Global Public Health, New York University, New York, USA; 6 Department of Epidemiology, School of Global Public Health, New York University, New York, USA; 7 Ophthalmology, Department of Surgery, University of Melbourne, Melbourne, Australia

**Keywords:** Food insecurity, COVID-19, Households with children, Social media

## Abstract

**Background:**

In the United States, approximately 11% of households were food insecure prior to the COVID-19 pandemic. The present study aims to describe the prevalence of food insecurity among adults and households with children living in the United States during the pandemic.

**Methods:**

This study utilized social media as a recruitment platform to administer an original online survey on demographics and COVID-related food insecurity. The survey was disseminated through an advertisement campaign on Facebook and affiliated platforms. Food insecurity was assessed with a validated six-item United States Department of Agriculture (USDA) Household Food Security Survey Module, which was used to create a six-point numerical food security score, where a higher score indicates lower food security. Individual-level participant demographic information was also collected. Logistic regressions (low/very-low compared with high/marginal food security) were performed to generate adjusted odds ratios (AOR) and 95%CIs for food insecurity and select demographic characteristics.

**Results:**

Advertisements reached 250,701 individuals and resulted in 5,606 complete surveys. Overall, 14.7% of participants self-identified as having low or very low food security in their households, with higher prevalence (17.5%) among households with children. Unemployment (AOR:1.76, 95%CI:1.09–2.80), high school or lower education (AOR:2.25, 95%CI:1.29–3.90), and low income (AOR[$30,000-$50,000]:5.87, 95%CI:3.35–10.37; AOR[< $30,000]:10.61, 95%CI:5.50–20.80) were associated with higher odds of food insecurity in multivariable models among households with children (and the whole sample).

**Conclusions:**

These data indicate exacerbation of food insecurity during the pandemic. The study will be instrumental in guiding additional research and time-sensitive interventions targeted towards vulnerable food insecure subgroups.

## Background

Household food insecurity is defined as the lack of consistent physical and economic access to sufficient, safe and nutritious foods for an active and healthy lifestyle [1]. Prior to COVID-19, approximately 11% of households in the United States (US) were food insecure; among them, 11 million children (7.8% of children nationwide) experienced food insecurity [2]. One in 7 children (15.2%) do not have consistent access to a nutritionally-adequate diet [3]. Furthermore, the “meal gap”, or shortfall in meals to adequately feed the population, was estimated at 7 billion a year before the pandemic [4]. Food insecurity is estimated to increase sharply due to COVID-19 [5, 6].

The pandemic has significantly changed the food environment. Higher unemployment due to pandemic-related job losses combined with interruptions in the food supply chain have left consumers to purchase foods that they can afford and access [7]. Lockdown measures have generally made it difficult to purchase perishable foods, particularly fresh produce [8, 9]. Perishable foods have been diminishing from the food supply. The supply chain of non-perishable and canned goods has also experienced delays due to a limited labor force and additional quality checks [7, 10]. The unpredictable food supply has led to consumers stockpiling shelf-stable foods, in particular foods that were accessible and affordable [5, 9]. Early reports during the lockdown from US grocery stores have noted historically high sales of shelf-stable and packaged ultra-processed foods [11, 12].

It is noteworthy that approximately 30 million school children depend on the National School Lunch program for low-cost or free lunches [13], and with school closures, new groups of children have become vulnerable to food insecurity. A recent study from Italy during the pandemic lockdown reported that children were eating more snack foods [14]. While there is currently no known US study addressing children’s eating behaviors during the pandemic, it has been reported that diets of the majority of American children from low-income families and from the population at large are inadequate and poor in dietary quality, particularly with regards to the intake of fruits, vegetables, refined grains, and seafood [15, 16]. The anticipated increase in the prevalence of food insecurity during the continually worsening COVID-19 epidemic in the US necessitates focusing on making healthful foods available for at-risk populations, including low-income households with children [5, 17]. Food insecurity is closely linked with obesity due to the consumption of energy-dense, nutrient-poor foods, particularly highly processed foods [18–21], a link which has been identified as a top research priority by the National Institutes of Health (NIH) [21]. Regions in the US that have the highest prevalence of food insecurity also record the highest prevalence of obesity [20]. Obesity has now been identified as a risk factor for COVID-19 severity [22].

Assessing prevalence of food insecurity nationwide is critical during the pandemic. We utilized social media as a recruitment platform to conduct studies related to food insecurity and the food environment in the US. Using validated measures from the United States Department of Agriculture (USDA), the present study aims to assess the prevalence of food insecurity in a nationwide sample of adults and of households with children during the COVID-19 epidemic in the US. These findings will be instrumental in identifying new groups of vulnerable individuals in need of tailored interventions to enhance the nutritional status of these subpopulations and to prevent obesity and its related long-term consequences.

## Methods

### Participant recruitment and analytic dataset

The recruitment and data collection strategy has been described elsewhere [23]. Briefly, an anonymous non-probability sample of social media users on Facebook, Instagram, Messenger, and the Facebook Audience network (other mobile apps and websites partnered with Facebook) was recruited by conducting an advertisement campaign with the link to an online Qualtrics (Provo, UT) survey. Recruitment occurred from April 16 to April 21, 2020; the advertisement campaign targeted adults aged ≥ 18 years of any sex residing in the US. Additional measures in the advertisement distribution were taken to oversample men as well as racial and ethnic minorities [23]. We conducted a comparison of participants collected through this methodology with US census data, which is presented in great detail in our methods paper [23]. Briefly, compared to the 2018–2019 US census data, we observed a relatively similar gender distribution (57.7% vs. 51.3% female), a lower proportion of younger adults (9.3% vs. 21.3% 18–29 year olds), a higher portion of Non-Hispanic whites (92.3% vs. 60.4%), and a lower proportion of those with high school or below educational attainment (15.7% vs. 39.5%) [23]. Eligibility was assessed using two screening questions to confirm that participants resided within the US and were ≥ 18 years. Those who completed the survey or were ineligible to participate were provided with a list of websites with COVID-19 resources from the World Health Organization (WHO) and the US Centers for Disease Control and Prevention (CDC). In order to minimize the potential for multiple respondents from the same household answering survey questions, a survey could not be submitted from the same Internet Protocol (IP) address. All study materials and procedures underwent Human Subjects Research review by the Institutional Review Board and were determined to be exempt (IRB-FY2020–4285).

### Data collection

The development of the survey questions was informed by past questionnaires on infectious disease outbreaks, principles of the Health Belief Model (HBM) [24–27], and the WHO tool for behavioral insights on COVID-19 [28]. Demographic questions included respondent self-reported sex, race, age, employment status, educational attainment, whether the respondent lived with children < 18 years of age, state of residence (re-coded by US Census region), urban/suburban/rural residence, marital status, and annual household income.

Food insecurity was assessed using the six-item USDA Household Food Security Survey Module, which was used to create a six-point validated numerical food insecurity score [29, 30]. The US began to experience the negative social and economic impact associated with the pandemic, in February 2020 [31]. Therefore, participants were surveyed in mid-April and asked about food insecurity concerns over the past three months. Answer choices were accordingly adapted to assess food insecurity in the last 3 months (an approximate start of the pandemic’s spread in the U.S). The scores in this survey range from 0–6. Households with scores ranging from 0–1 were considered to have “*High to marginal levels of food security,”* defined as having consistent access to sufficient variety, quantity and quality of food either all or most of the time, respectively. The USDA combines high or marginal food security into a singular category [29]. Scores from 2–4 were categorized as the *“Low Food Security”* group, characterized by sufficient caloric intake or quantity of food, but with compromised diet quality; and scores from 5–6 were categorized as the *“Very Low Food Security”* group, in which one or more members of the household experience disruptions in food intake; food quality and quantity are compromised, and the household experiences a lack of food, or resources to obtain it. In order to align with the precedent on past utilization of the USDA scale [32], including during COVID-19 [33], the food insecurity scores were analyzed categorically based on the USDA defined thresholds of food insecurity. Two questions addressing additional dimensions of food insecurity were included by the study team: 1) Because I need food to eat, I have eaten packaged foods that have expired dates or that are past the "best by dates”, and 2) “Because I need food to eat, I have eaten perishable food such as fruits and vegetables that do not appear to be fresh.”

### Analytic sample

The social media advertising was able to reach 250,701 individuals and resulted in a total of 6,676 responses, of which 6,518 met the survey criteria and commenced the survey. Of these participants, 5,606 had data on food security, and 1,452 (25.9%) identified living with children < 18 years of age (Fig. 2). Four individuals (0.18%) who did not identify as either male or female were not included in the analyses because of low numbers. For the regression analyses, participants with missing values for relevant socioeconomic variables were excluded (n = 1,294). A total sample of 4,312 participants were retained in the logistic regression models.

### Statistical analysis

**Fig. 1:**
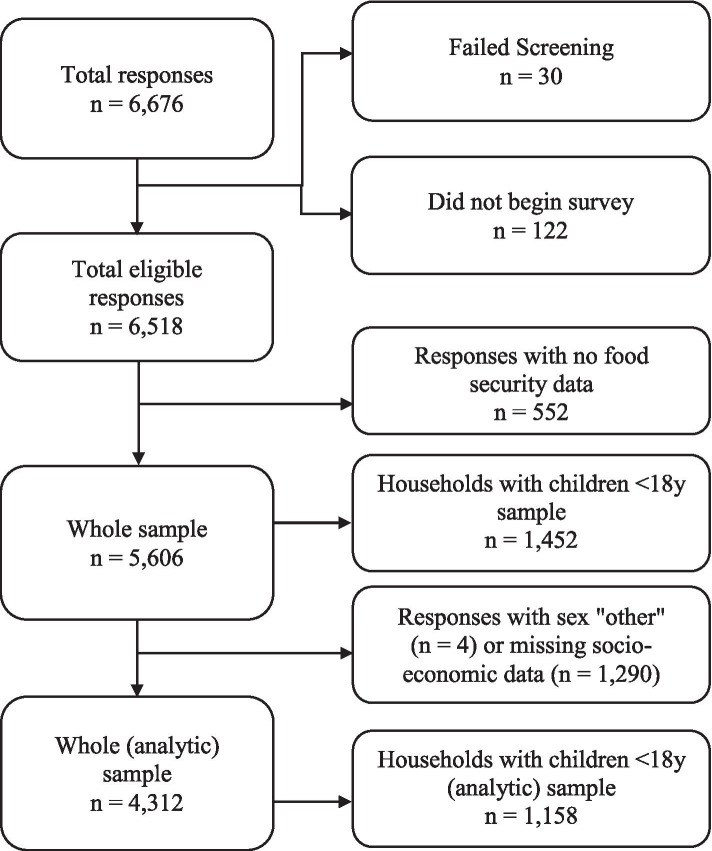
Final analytic sample derived from the total responses received during social media data collection

For the purposes of these analyses we combined the two categories that indicate food security, namely “High Food Security” and “Marginal Food Security” into “High/Marginal Food Security”, and food insecurity, namely "Low Food Security" and "Very Low Food Security" (Fig. 1). Descriptive statistics of participant characteristics were computed by the three categories of food insecurity (High/Marginal Food Security; Low Food Security; Very Low Food Security). A multivariable logistic regression was conducted to assess odds of low or very low food security (compared to high/marginal food security) by the socio-demographic predicator variables for the whole sample and for households living with children < 18 years. The covariates in the adjusted models (sex, age, race, region, urban/suburban/rural residence, employment status, education, marital status, income, and number of people in the household) were selected based on past evidence of socio-economic predictors of food insecurity from the literature [34, 35].

## Results

### Participant characteristics

In both the whole sample and households with children, most participants were female (58.5% in the whole sample, 62.2% among households with children), Non-Hispanic white (92.9% in the whole sample, 91.7% among households with children), and employed (56.3% in the whole sample, 70.3% among households with children), married or cohabiting (74.1% in the whole sample, 84.6% among households with children), and living in suburban environments (53.3% in the whole sample, 59.2% among households with children) (Table 1). Likewise, most participants reported living with 3 to 4 people (65.8%) in the household during the pandemic (data not shown). The majority of participants received a food insecurity score reflecting high or marginal food security (whole sample: 85.3%; households with children: 82.5%;), followed by low food security (whole sample: 8.7%; households with children: 10.3%), and very low food security (whole sample: 6.0%; households with children: 7.2%).

**Table 1:** Characteristics of 5,606 participants with data on household food insecurity in online COVID-19 survey

Variable	Whole sample					Households with children < 18YRS				
	**All** **n = 5,606**	**HFS n = 4,781**	**LFS** **n = 486**	**VLFS** **n = 339**	*p*-value*	**All** **n = 1,452**	**HFS** **n = 1,198**	**LFS** **n = 149**	**VLFS** **n = 105**	*p*-value*
**Sex (%)**					0.081					0.068
Female	58.5	57.9	62.6	61.1		62.2	60.9	69.9	66.7	
Male	41.5	42.1	37.4	38.9		37.8	39.1	30.1	33.3	
**Age (%)**					< 0.001					0.045
18–38 years old	16.5	15.4	23.3	22.7		31.3	29.5	38.3	41	
40–59 years old	43.7	43.1	44.4	51.3		59.6	61.2	53	50.5	
60 + years old	39.8	41.5	32.3	26.0		9.2	9.3	12.8	14.3	
**Race (%)**					0.001					0.004
White, Non-Hispanic	92.9	93.4	90.7	88.5		91.7	92.8	87.2	85.7	
Non-White^a^	7.1	6.6	9.3	11.5		8.3	7.2	12.8	14.3	
**Region (%)**					0.306					0.981
Northeast	27.3	26.9	30.8	27.3		37.7	27.6	29.5	25.8	
Midwest	25.8	26.4	21.2	24.7		37.9	28	24.8	30.1	
South	27.3	27.0	28.2	28.9		34.9	24.9	24.8	25.8	
West	19.6	19.7	19.8	19.1		19.5	19.5	20.9	18.3	
**Residence (%)**					< 0.001					0.107
Suburb	53.3	54.3	50.3	43.4		59.2	60.8	53.5	49.5	
Urban	15.2	14.4	19.4	20.4		10.6	10	11.6	16.1	
Rural	31.5	31.3	30.3	36.2		30.2	29.2	34.9	34.4	
**Employment (%)**					< 0.001					< 0.001
Employed	56.2	57.8	48.3	45.1		70.3	73.3	55	57	
Student/Unpaid work	5.5	5.0	7.7	9.5		12.4	11.2	17.1	19.4	
Not Work./Unemp	12.5	9.5	28.9	32.2		11.8	9.4	26.4	20.4	
Retired	25.7	27.7	15	13.2		5.5	6.1	1.6	3.2	
**Marital status (%)**					< 0.001					< 0.001
Married/cohabiting	74.1	74.9	69.3	69.0		84.6	87.7	73.6	64.5	
Single	25.9	25.1	30.7	31.0		7.0	5.4	13.2	17.2	
Divorced/separated/widowed	12.7	11.5	16.2	24.3		8.4	7.0	13.2	18.3	
**Education (%)**					< 0.001					< 0.001
High School or less	10.3	9.1	16.1	19.0		9.8	7.9	16.5	21.7	
Some Col. / Assoc. D	34.2	32.8	40.5	45.3		30.7	28.5	38.6	45.7	
Bachelor’s D. +	55.5	58.1	43.4	35.7		59.5	63.6	44.9	32.6	
**Income (%)**					< 0.001					< 0.001
Less than $30,000	13.4	9.1	31.6	47		8.2	4.3	19.0	37.2	
$30,000 to less than $50,000	15.5	13.8	26.5	22.6		10.6	8.2	22.4	22.1	
$50,000 to less than $75,000	17.7	18.1	17	13.3		15.3	15.0	19.0	14.0	
$75,000 to less than $100,000	20.8	22.2	13.3	11.1		22.2	23.2	18.1	16.3	
$100,000 or more	32.7	36.8	11.7	5.9		43.7	49.3	21.6	10.5	

*Suburb* Suburban, *Not Work/Unemp* Not working or Unemployed, *Som Col./ Assoc. D* Some college or Associates Degree; Bachelor's D. + : Bachelor's degree or above

^a^Non-white race included Asian/Pacific Islander, Black, Non-Hispanic, Hispanic/Latinx, Native American, multiracial or other,

HFS: High/Marginal Food Security; LFS: Low Food Security: VLFS: Very Low Food Security

^*^p-values calculated from Chi-squared tests

### Prevalence of food insecurity

**Fig. 2:**
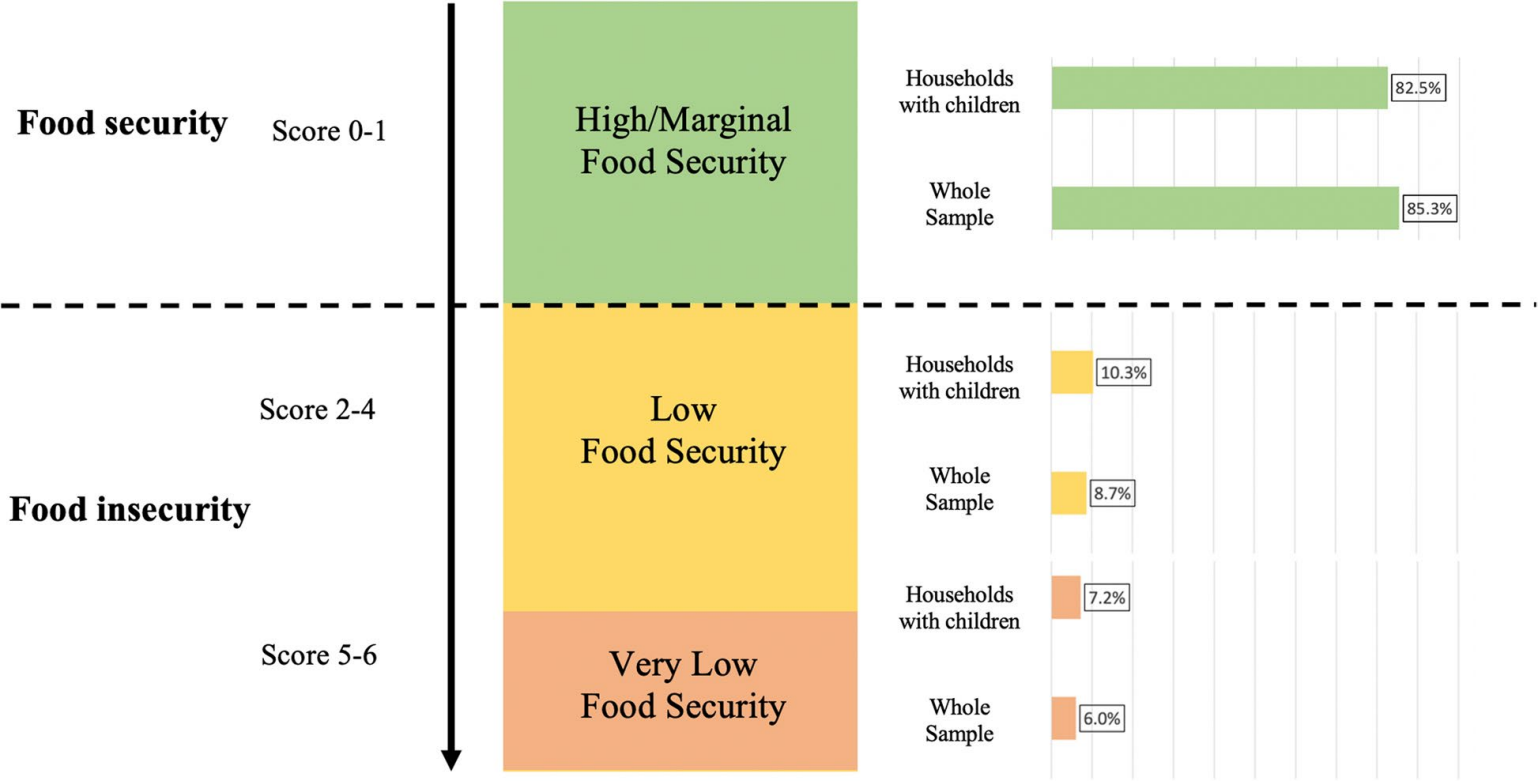
Food insecurity among whole sample (n = 5,606) and households with children < 18yrs (n = 1,452) based on USDA-defined [29, 30] food security categories

Overall, 825 participants (14.7%) in the whole sample displayed low (8.7%) or very low (6.0%) levels of food security, while 254 participants (17.5%) among those with children displayed low (10.3%) or very low (7.2%) levels of food security (Fig. 2).

Prevalence of self-reported food insecurity were computed from the responses to the USDA assessment tool [29] and are displayed in Table 2. Among the six USDA food insecurity questions, the issue with the highest proportion of participants selecting the response “Often true” or “Sometimes true” was not being able to afford to eat balanced meals (16.7% in the whole population, 19.2% among households with children). Furthermore, 23.0% of households from the whole sample and 24.1% percent of households with children reported consuming packaged foods that had passed expiration dates. Of the participants who had to reduce the portion size of their meals or skip meals due to lack of money or food in the last 3 months (10.9% in whole sample, 13.7% among households with children), a significant proportion of participants noted that this occurred almost every week (22.5% in whole sample, 23.6% among households with children). The proportion of individuals reducing the portion of their meals due to lack of money or food was also noteworthy (11.2% in whole sample, 13.6% among households with children), as was the proportion who could not eat despite being hungry due to lack of money or food (8.2% in whole sample, 10.5% among households with children).

**Table 2:** Household food insecurity-related attributes among 5,606 study participants

**Food security survey**	**% (Whole sample)** **n = 5,606**	**% (HH without child < 18)** **n = 4,154**	**% (HH with child < 18)** **n = 1,452**	*p*-value*
**In the last 3 months, the food that (I/we) bought just didn’t last, and (I/we) didn’t have money to get more**				0.055
Often true	2.2	1.9	2.9	
Sometimes true	10.3	9.9	11.4	
Never true	84.6	85.2	82.7	
Don't know / Prefer not to say	2.9	2.9	3.0	
**In the last 3 months: (I/we) couldn’t afford to eat balanced meals**				0.010
Often true	4.5	4.1	5.6	
Sometimes true	12.2	11.7	13.6	
Never true	81.7	82.4	79.5	
Don't know / Prefer not to say	1.7	1.8	1.3	
**In the last 3 months: Because I need food to eat, I have eaten packaged foods that have expired dates or that are passed the "best by" dates**				0.516
Often true	3.1	3.2	3.0	
Sometimes true	19.9	19.5	21.1	
Never true	74.7	74.9	73.9	
Don't know / Prefer not to say	2.3	2.4	2.1	
**In the last 3 months: Because I need food to eat, I have eaten perishable food such as fruits and vegetables that do not appear to be fresh**				0.041
Often true	2.3	2.1	2.8	
Sometimes true	16.6	16.8	16.0	
Never true	79.6	79.4	80.3	
Don't know / Prefer not to say	1.5	1.7	0.9	
**In the last 3 months: Did you or other adults in your household ever cut the size of your meals or skip meals because there wasn't enough money for food?**				< 0.001
Yes	10.9	10.0	13.7	
No	89.1	90.0	86.3	
*How often did this happen?*				0.468
Almost every week	22.5	22.0	23.6	
Some weeks but not every week	42.1	44.2	37.7	
Only 1 or 2 weeks	25.8	24.9	27.6	
Don't know	9.6	8.9	11.1	
**In the last 3 months: Did you ever eat less than you felt you should because there wasn't enough money for food?**				0.004
Yes	11.2	10.4	13.6	
No	87.0	87.8	84.7	
Don't know	1.8	1.8	1.7	
**In the last 3 months: Were you ever hungry but didn't eat because there wasn't enough money for food?**				0.001
Yes	8.2	7.4	10.5	
No	90.7	91.5	88.3	
Don't know	1.1	1.1	1.2	

*HH* Households; *p-values calculated from chi-squared tests

### Determinants of food insecurity

Table 3 describes the socio-demographic determinants observed to be associated with food insecurity among the study sample. Overall, in comparison to the whole sample, households with children < 18 years of age had significantly higher odds of food insecurity (adjusted odds ratios [AOR]:1.64, 95% CI:1.27–2.12) when adjusted for other socio-economic variables. We report that in the whole sample and those living with children, participants who were not working or unemployed had a higher odds of being food insecure (having low or very low food security) compared to those who were employed (AOR[whole]: 2.19, 95% CI:1.78–2.78; AOR[hh-child.]: 1.76, 95% CI: 1.09–2.80). Participants reporting a high school degree or lower similarly had higher odds of being food insecure than those with a Bachelor’s degree or higher in both groups (AOR[whole]: 1.51, 95% CI: 1.13–2.02; AOR[hh-child]: 2.25, 95% CI: 1.29–3.90). Among those with children, compared to those with a household annual income of $100,000 or more, those with incomes between $75,000-$100,000, $50,000-$75,000, $30,000-$50,000, or less than $30,000 had progressively higher odds of being food insecure (AOR: 2.05, 95% CI: 1.21–3.49; AOR: 2.83, 95% CI: 1.63–4.91; AOR: 5.87, 95% CI: 3.35–10.37; AOR: 10.61, 95% CI: 5.50–20.80). Living in the Midwest was associated with 31% lower odds of food insecurity as compared to the Northeast but only in the whole sample (not in the subset of households with children), and living in urban versus rural areas was not found to be associated with food insecurity in this sample.

**Table 3:** Adjusted Odds of low or very low food security among 4,312 participants with data on household food insecurity

Variable	Whole sample	Households with children < 18y
**Race (%), n = 1,452 **^**a**^		
White, Non-Hispanic	Ref	Ref
Non-White	1.32 (0.92–1.85)	1.72 (0.91–3.17)
**Region (%), n = 1,300 **^**b**^		
Northeast	Ref	Ref
Midwest	**0.69 (0.52–0.90)**	0.81 (0.51–1.30)
South	0.97 (0.75–1.24)	0.93 (0.57–1.51)
West	(0.78 (0.59–1.04)	0.79 (0.46–1.35)
**Residence (%), n = 1,300 **^**c**^		
Suburban	Ref	Ref
Urban	1.27 (0.98–1.65)	1.42 (0.80–2.43)
Rural	0.91 (0.73–1.12)	0.94 (0.64–1.38)
**Employment (%), n = 1,300 **^**d**^		
Employed	Ref	Ref
Student/Unpaid work	1.27 (0.85–1.86)	1.54 (0.92–2.55)
Not Work./Unemp	**2.19 (1.78–2.78)**	**1.76 (1.09–2.80)**
Retired	**0.57 (0.42–0.78)**	**0.21 (0.05–0.65)**
**Marital status (%), n = 1,300 **^**e**^		
Married/cohabiting	Ref	Ref
Single	1.04 (0.81–1.34)	1.34 (0.72–2.43)
Divorced/separated/widowed	1.14 (0.86–1.50)	1.41 (0.76–2.58)
**Education (%), n = 1,290 **^**f**^		
Bachelor’s D. +	Ref	Ref
Some Col. / Assoc. D	**1.25 (1.02–1.54)**	1.42 (0.95–2.10)
High School or less	**1.51 (1.13–2.02)**	**2.25 (1.29–3.90)**
**Income (%), n = 1,168 **^**g**^		
$100,000 or more	Ref	Ref
$75,000 to less than $100,000	**2.41 (1.69–3.46)**	**2.05 (1.21–3.49)**
$50,000 to less than $75,000	**3.71 (2.61–5.30)**	**2.83 (1.63–4.91)**
$30,000 to less than $50,000	**7.61 (5.41–10.83)**	**5.87 (3.35–10.37)**
Less than $30,000	**16.46 (11.39–24.07)**	**10.61 (5.50–20.80)**

*Suburb*. Suburban, *Not Work/Unemp.* Not working or Unemployed, *Som Col. / Assoc. D*. Some college or Associates Degree, *Bachelor's D.* + Bachelor's degree or above

^a^ Adjusted for sex, age, region, residence, employment, marital status, education, income, number of people in household

^b^ Adjusted for sex, age, race, residence, employment, marital status, education, income, number of people in household

^c^ Adjusted for sex, age, race, region, employment, marital status, education, income, number of people in household

^d^ Adjusted for sex, age, race, region, residence, marital status, education, income, number of people in household

^e^ Adjusted for sex, age, race, region, residence, employment, education, income, number of people in household

^f^ Adjusted for sex, age, race, region, residence, employment, marital status, income, number of people in household

^g^ Adjusted for sex, age, race, region, residence, employment, marital status, education, number of people in household

## Discussion

In this nationwide study assessing food insecurity during the COVID-19 pandemic using a convenience sample of US social media users, we observed that approximately 15% of households experienced either low or very low food security, of which 8% reported very low food security. Despite surveying a convenience sample of social media users with higher socioeconomic status compared to the general US population [23, 36], higher prevalence of food insecurity was reported compared to pre-pandemic national prevalence data [30]. In this study, demographic factors that were the strongest determinants of food insecurity were unemployment, education (less than a Bachelor’s degree), and lower income. These associations persisted, and were stronger, among households with children, where 17.5% experienced food insecurity. Our study observed that households in the Midwest had the lowest odds of being food insecure, which is also consistent with reports indicating lower food insecurity prevalence than the national average for this region [30]. Our study results are consistent with early reports of potential increases in food insecurity during the pandemic in the overall population, and in households with children [37]. Preliminary data from a national survey showed that 20 states have higher prevalence of food insecurity as compared to pre-pandemic prevalence data, although more detailed analyses are forthcoming [6, 38].

It is well-known that food insecurity is associated with poor dietary quality and nutrient-poor, energy-dense foods, such as sugary beverages as well as salty and high-fat foods [20]. Even prior to the COVID-19 pandemic, the diets of most American children, regardless of socio-economic status, failed to meet national dietary recommendations [15]. A pre-pandemic report describing the diets of children and adolescents younger than 20 years of age noted that approximately 15% of their caloric intake was obtained from added sugars, primarily from sugary beverages, fruit drinks, sweet bakery products, candy, cereals, and frozen dairy products [38]. This comes against an alarming backdrop of a doubling of caloric contribution from salty snack foods over the past four decades, particularly among Non-Hispanic Blacks and children from low-income households [15, 39]. A study done in Italy during the lockdown reported that while no changes were observed in fruit and vegetable intake, consumption of potato chips, red meat, and sugary drinks increased significantly during this period [14]. Another study during the pandemic in Italy showed a shift in eating patterns, with approximately one third of the population reporting eating less healthy foods [40]. The mandated pandemic lockdowns played a role in exacerbating food insecurity; we speculate that supply chain disruptions resulted in inadequate inventory for smaller grocery stores, and food distribution centers were overwhelmed, thereby depriving communities of fresh produce. Moreover, lockdown measures also disrupted food purchasing behaviors (e.g., concerns about in-grocery store shopping) [41]. Taken together, the results of our study and other reports [8] suggest that COVID-related food insecurity has contributed to an evolving public health crisis.

The present study also explored the utility of social media outlets as a platform for participant recruitment for studies addressing food security at the population-level. To this end, our study supports the cost efficiency (< 18 cents per respondent) of this method [23]. Approximately 70% of American adults use Facebook [42], and the Facebook platform enabled us to reach a large, geographically diverse sample in a short period of time through social media advertisements, including vulnerable sub-populations during times when in-person recruitment is unsafe and not permitted by institutional review boards. While the authors recognize that this is not a representative sample, we highlight that social media-based recruitment is a powerful strategy to rapidly collect health-related data during emerging health crises such as COVID-19 when traditional data collection opportunities are limited [23] and will likely remain as an integral strategy for recruitment into epidemiologic studies. Facebook has been extensively utilized in previous health research and shown to be a valid, low cost, and efficient recruitment tool as documented in a systematic review of 35 studies [43] and in our published COVID-19 research [44–46]. However, there were some challenges with social media recruitment. Although the research team was vigilant and closely monitored the demographic composition of the sample, it was not possible to override the Facebook algorithms for targeted advertising that sent the survey to participants who were identified as likely responders to achieve the lowest average cost per link click across the various platforms [23]. In an attempt to mitigate the biasing effect of Facebook’s algorithms, to increase the participation of respondents with specific sociodemographic characteristics, the study team implemented tailored compensatory advertisements, for example to increase the proportion of men in the study [23].

The study provided some important perspectives regarding public health disparities with regards to food insecurity during a public health crisis. The study strengths included: 1) quickly reaching a large, geographically diverse sample of US adults during the height of the COVID-19 pandemic, and 2) employing an established, validated food insecurity instrument commissioned by the USDA to provide a relatively comprehensive picture of food insecurity among study participants. However, some limitations of this study must be considered. The sampling strategy resulted in a convenience sample of Facebook users (~ 99%) and other Facebook platforms (~ 1%). The non-generalizability of findings due to higher incomes (74% of Facebook users are high-income earners [42]) and over-representation of Non-Hispanic whites, specifically women, must be acknowledged. We acknowledge that the subgroups of young adults, racial and ethnic minorities, and those with lower educational attainment (often an indicator of lower socio-economic status) were less represented in our study sample, who generally experience higher rates of food insecurity [8, 30, 47, 48], therefore confirmation in additional studies is needed that are better able to decipher associations in those specific subgroups.

The present study does not contain data on specific household food purchases. Subsequent waves of data collection will assess food purchasing behaviors with respect to specific foods such as snack foods, sugary beverages, cereals, and other shelf-stable foods, as well as the use of food assistance programs. Although some regional disparities in food-insecurity were also identified in analyses, further localized policy analyses as well as in-depth mixed-methods research will provide evidence to identify reasons behind region-based disparities in food insecurity during the COVID-19 pandemic. Questions on the use of the US Supplemental Nutrition Assistance Program (SNAP), the specialized program for Women, Infants, and Children (WIC), or the use of other supplemental food sources (e.g. food pantries or school lunch programs) should also be explored in further research.

### Research and public health implications

Our study has pin-pointed the need for additional quantitative research including in-depth and potentially representative analyses in this underserved sub-population and their experiences with food insecurity, and qualitative research to identify the factors contributing to the rise in food insecurity during the pandemic among households with children who, in our sample, were predominantly white with higher levels of education and income compared to the general US population.

From a public health policy and practice lens, this study has important implications for public health interventions to alleviate food insecurity through legislative changes to prevent a further surge in food insecurity and its subsequent consequences. *Food policy changes* are essential and may include further changes to expand SNAP and WIC benefits, including waivers on the SNAP time limit for unemployed individuals [49]. Protectionist and food nationalism policies to promote local partnerships between farmers and food distribution networks as well as government incentives to farmers are key in building a resilient food system, and importantly one that is health promoting. Another avenue to consider is *food salvaging efforts* to reduce the meal gap. Stockpiling during the pandemic is expected to result in immense food waste from lack of planning, improper storage, and inability to consume foods in a timely manner. Food salvaging efforts and reduction of food waste to divert as meals for families is another avenue where public health policy can impactfully reduce food insecurity. Digital marketplaces and mobile technology can help scale up such efforts [50]. *Nutrition literacy* interventions (defined as the competence and skills necessary to critically assess food and nutrition information which people are exposed to) and related *media literacy* (defined as the cognitive ability to recognize and evaluate media messaging around foods) are two overlooked strategies to reduce food insecurity [50]. Moreover, health professionals must emphasize the avoidance of highly processed, unhealthy shelf-stable foods, taking into consideration food access or financial constraints faced by individuals, food banks and community food programs, which have worsened during the pandemic. They can work collaboratively with food banks and community feeding programs to ensure that the foods distributed are nutrient dense.

Finally, and importantly, food insecurity is associated with obesity; although there is no conclusive evidence, higher prevalence of food insecurity during the pandemic is also a threat to increasing the prevalence of obesity post-pandemic [51]. For example, in New York City, Bronx has the highest prevalence of hunger coupled with a high prevalence of obesity and cardiovascular risk factors [20, 52], and is a community at risk for obesity-related COVID-19 complications [22]. Therefore, it is imperative to flatten the food insecurity curve in parallel to the COVID-19 curve as a strategy for *obesity prevention* in vulnerable communities.

## Conclusion

Food security is a basic right for all individuals and the persistent prevalence of food insecurity is more than a health problem [53]. Our data suggest that there has been heightened food insecurity during the COVID-19 pandemic. Additional qualitative and quantitative research is warranted based on the findings of our study. The exacerbated prevalence of food insecurity may contribute to excess weight, which may relate to co-morbidities and chronic diseases. Therefore, timely interventions to avert food insecurity are urgent. The continued COVID-19 pandemic emphasizes the need for multi-level approaches to address food insecurity. As we continue to adapt to this evolving public health crisis, we know that while many effects of food insecurity are immediate, some are long-term. Strategies to tackle food security must be considered as an important part of emergency preparedness planning efforts that are underway.

## Data Availability

The datasets used and/or analyzed during the current study are available from the corresponding author on reasonable request.

## Abbreviations

US: United States
USDA: United States Department of Agriculture
WHO: World Health Organization
CDC: US Centers for Disease Control and Prevention
HBM: Health Belief Model
AOR: Adjusted Odds Ratio
SNAP: Supplemental Nutrition Assistance Program
